# Farmland Transfers in China: From Theoretic Framework to Practice

**DOI:** 10.3390/ijerph19010217

**Published:** 2021-12-25

**Authors:** Menglin Ou, Jian Gong

**Affiliations:** School of Public Administration, China University of Geosciences, Wuhan 430074, China; 201810297@cug.edu.cn

**Keywords:** land use management, sustainable development, rural revitalization strategy, review

## Abstract

Land transformation in agriculture is a crucial global issue for food safety and regional sustainable development. In the context of Chinese rural revitalization strategy, farmland transfer has become an increasingly engaging area of focus for those in a broad range of fields. In this paper, we make a comprehensive review of land transformation in agriculture through literature analysis. Farmland transfers in China were characterized as five dimensions: public policy, market mechanisms, influencing factors, optimization of spatial distribution, and practical results. Meanwhile, we shed light on limitations of the theories and methodologies for farmland transfers in previous studies, and propose the highlights of farmland transfers in China in the future: (1) refining the theoretical systems of farmland transfer under the background of transformations, (2) optimizing land use configuration for farmland transfers within the context of national strategic decisions, (3) developing the land use model supported by big data for understanding farmland transformation; (4) enhancing the comprehensive analysis and interdisciplinary application perspective for farmland transfer issues.

## 1. Backgrounds

Farmland transfer is a key factor affecting agricultural sustainable development in China. It is defined as the transfer of farmland contractual management rights via rental, exchange, or subcontracting, following the law, characterized by the change in scale, means, and uses of farmland in The Rural-Land Contract Law of the People’s Republic of China. With the improvement of market economics in China and the preliminary establishment of a new type of agricultural management, farmland transfer plays a key role in agricultural modernization, industrialization, and intensification. The transfer of farmland contractual management rights has become a definite trend for promoting agricultural development [1]. Now, China is at a critical juncture of social transformation. Studies of farmland transfer are more relevant, diverse and in-depth due to the implementation of rural area revitalization, targeted poverty alleviation, and other strategies [2,3]. Furthermore, the research focus of farmland transfer evolves with every change in a social and economic context and the resulting reform of the farmland system. Therefore, it is crucial to figure out the future development trend and research focus of farmland transfer in China.

To provide useful support for further research on this subject, this paper collected, categorized, collated, and summarized existing findings on farmland transfer in China. Based on the literature analysis, with timeline, of this issue, this paper analyzed and characterized farmland transfer in China as five dimensions, namely, public policies, market mechanism, influencing factors, optimization of spatial distribution, and practices of farmland transfer. The main objective is to suggest possible priorities for future research on farmland transfer in China, thus providing direction for related studies.

## 2. Developments of Farmland Transfer in China

**Figure 1:**
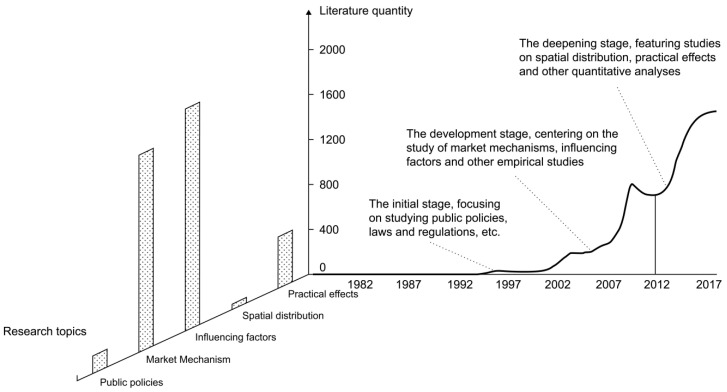
3D chart of farmland transfer literature.

Several studies on farmland transfer in China have developed, yielding findings about China’s farmland system. With the adoption of the Open Door Policy in 1978, rural households could own farmlands by contract and obtain the contractual land rights. In this period, farmland transfer started to take shape in China. According to the reform of the farmland system and changes in the social and economic context, the research topics of farmland transfer varied among several stages. The research methodology of farmland transfer has changed from qualitative to quantitative analysis, and the research perspective has changed from pertaining to a single discipline to a cross-discipline one, yielding extensive and diverse research results (Figure 1). Combining the evolution of research implications, themes and contentious issues of farmland transfer, this paper divided the timeline of relevant studies into three phases by retrieving and analyzing the literature.

### 2.1. The Initial Stage (from the Early 1980s to 2001)

During this stage, most studies were related to policies and legal matters and focused on the formulation of policies, laws, and regulations on farmland transfer. In the 14th National People’s Congress of the Communist Party of China (CPC) in 1992, it was posited that the reasonable transfer of farmland should align with the objective model of the socialist market system, and it should be developed on the basis of a stable household responsibility system. The 14th National People’s Congress also pointed out that not only measures should be taken to improve the macro context of agricultural production nationwide, but also to fine-tune and transition the land system arrangement to suit the social and economic climate. In this stage, most studies (e.g., Qu et al. [4] and Gengaje [5]) focused on the analysis of the farmland transfer system, laws, and regulations and expanded gradually to discussions about the formulation, development, and evolution of public policies on farmland transfer and related contents.

### 2.2. The Development Stage (from 2002 to 2011)

During this stage, empirical studies on establishing the market mechanisms and analyzing the influencing factors for farmland transfer were important issues. With the deepening of farmland system reform and the development of new types of urban–rural relationships, farmlands played a critical role as a means of production. But the economy of scale and intensive production were difficult to realize under the constraints of the small scale of farmlands individually operated by households. Additionally, land use policies exerted a determining effect on the development of the farmland transfer market, which was also constantly improving [6,7]. Therefore, related studies began to pay attention to the role of market mechanisms (e.g., Jia et al. [1] and Ye et al. [6]) and the influencing factors of farmland transfer (e.g., Huang et al. [8] and Chen et al. [9]). The research schemes were dominated by micro-analysis and survey interview at the farm household scale.

### 2.3. The Deepening Stage (from 2012 to the Present)

This stage was characterized by comprehensive quantitative analysis such as the pattern optimization and practical effectiveness of farmland transfer. After the 18th National People’s Congress of the CPC in 2012, the agricultural cooperatives, leading agricultural industrialized enterprises and other new agricultural business entities have matured continuously [10,11]. Meanwhile, the launch of targeted alleviation and rural revitalization campaigns led to a new turn in the studies on farmland transfer. Farmland transfer decisions began to form their geographical and practical basis. Research topics on these two dimensions are characterized by cross-disciplinary and comprehensive features. The study of geographical characteristics has focused on how basic features of rural household labor, regional development, economic features of rural households, farmland resources of households, and other elements were reflected on geographical space and the figurative expression of related policies [12,13]. In contrast, studies on practices have focused on the comprehensive effects of public policies, market mechanisms, driving mechanisms, and transfer models of farmland transfer [14,15].

## 3. Reviews of Farmland Transfer in China

Farmland transfer studies in China have been characterized as five dimensions according to study focus, namely, public policies, market mechanisms, influencing factors, optimization of spatial distribution, and practical results. Studying the public policies on farmland transfer is conducive to grasping development trends and the direction of farmland transfer. Studies on market mechanisms aim to understand the approaches, scale, and direction of the farmland transfer market-based model [16,17,18]. The influencing factors of farmland transfer indicate its driving mechanism. Studies on the spatial distribution of farmland transfer reveal the spatial and temporal changes of the farmland transfer scale and rate [13,19]. Studying the practices of farmland transfer means determining the centralized display and concrete experience of farmland transfer models.

### 3.1. Farmland Transfer Public Policies

In China, there has been progressive exploration and analysis of farmland transfer. It started as a mechanism to allow development outside the prevailing system and policy. Then it gradually became a basic approach to agricultural development and for production mode transformation. Political system reform and the introduction of various public policies have established the basic conditions for farmland transfer in China. Since the 1990s, relevant decision-making authorities have announced a series of farmland reform policies (Table 1) aimed at promoting farmland transfer. Most of these important farmland reform policies were introduced during meeting sessions of the CPC. The decision-making bodies are the CPC central committee and the state council, the promulgation body is the standing committee of the National People’s Congress, or relevant government departments that issue detailed documents of implementation. From the collation of related literature and farmland policies at the national level, it can be observed that research on farmland transfer got updated with the announcement of public policies and relevant recommendations were made in a targeted manner. Especially since the 18th National People’s Congress of the CPC, the new policy on farmland transfer has been formed during the period of comprehensive deepening reform in China to better promote the development of rural areas, agriculture, and farmers.

Academic research provides arguments for specific laws, regulation, and public policies. In recent years, in-depth reform of the farmland system and the establishment of a new agricultural operation system have gradually improved the public policy system of farmland transfer and moderate scale operation. Therefore, scholars have started to interpret the implications of farmland transfer from the perspective of the farmland system and public policies. Pan and Chen [20] found that most farmland transfer activities in the early stage were voluntary and disorderly because of the lack of a corresponding land policy, system, and regulation. Wang [21] analyzed the role of the community organization system in promoting the farmland transfer and expanding the scale of land management to accommodate the rural productivity development in the context of rural reform. Farmland transfer is a necessary requirement to promote issues related to agriculture, rural areas, and farmers. The political barriers confronting effective farmland transfer need to be promptly addressed [22,23]. Based on a field study of land issues in many provinces, Jiang et al. [24] discussed the drawbacks of the duality of China’s land system, and posited that the land property tax distribution program was an important basis for ensuring farmers to be the main beneficiaries of land transfer. Xia [25] regarded “theoretical rights” as the core of the collective organization of villages, which autonomously made specific implementation plans based on the system to achieve the objectives of agricultural modernization for farmland scale management. In the early stages, farmland management adopted a singular legislation policy that only underlined the security function of the land. Later, rural-land legislation in China gradually followed and utilized the market rules, achieving a transition from singular to integrated policy. Nowadays, the transfer of land management rights is normalized from the perspective of real estate property rights mechanisms through laws, regulations, and public policies [26].

The evolution of the farmland transfer public policy system in China has been a process of normalization, and its content has been enriched. The basic content of modern agricultural development is the reconstruction of a new agricultural production model and agricultural business system, as well as the improvement of agricultural production efficiency and agricultural market activity on this basis. Practical farmland transfers need to be bound by public policies and regulations in order to achieve this goal. Research on this subject is an important reference for promoting the development of the land transfer public policy system in China and has paved the way for the emergence of studies on other related topics of land transfer.

### 3.2. Market Mechanism of Farmland Transfer

Farmland under the policy of private land ownership adopted by various countries can directly enter the market as a means of production. In contrast, China’s farmland system is dominated by collective ownership. This has shortcomings when it comes to establishing a farmland transfer market, including unclear ownership of the land and an underdeveloped farmland market. With the gradual improvement of the public policy system, the farmland transfer market started to operate with scale and in a centralized way. To cope with the land system of China, scholars have analyzed the market dynamics of farmland transfer in terms of market form, production cost and labor force conditions, and explored ways to regulate the farmland transfer market in China. Zhang [27] analyzed the land transfer cases in a cross-section between urban and rural areas and suggested that there were both market and non-market approaches to farmland transfer. Kung’s study found that farmland endowment and non-farm income opportunities resulted in the largest reduction of the farmland transaction cost [28]. It was not until the 2000s that the farmland transaction market gradually began developing in accordance with the development trajectory of the non-agricultural labor market [29]. The development of a farmland transfer market promoted farmland management rights and the scale of farmland operations, which were advantageous for the economies of scale and utilization efficiency of the farmland [30]. A realistic market for farmland transfer could only be formed when effective supply and demand coexist [31]. Jin and Deininger [32] adopted the farmer model to study the farmland transfer market of China and proved that the development of the farmland transfer market was conducive to the improvement of agricultural production rate.

Many researches focused on specific issues in the farmland transfer market of China. Feng et al. [33] found that the idle farmland issue due to labor migration was closely linked to the unsound market. Deng et al. [34] studied the issue from the perspective of key market components and suggested that the farmland transfer market had several pitfalls, including low prices, lack of driving force, slow market growth, poor agent service, and outdated market management. Wen [35] suggested that the main factors curbing the development of the farmland transfer market were insufficient safeguards, an unstable rural society, incomplete structuring of farmland rights, and irrational transfer rules. On this basis, scholars started to explore the decision-making behaviors of farmers in farmland transfer from micro-level perspectives. Based on the data from a multi-year national farm household survey, He et al. [36] found that the main players in the farmland transfer market were normal farmers. Other business entities participating in farmland transfers significantly increased in the past two years, and the transaction methods without agreement on the duration of transfer were also increasing [36]. There are some uncertainties in China’s farmland transfer market, mainly including the lack of social security and legal systems. In addition, the small market size, insufficient government intervention, severe market monopoly, overwhelming market information asymmetry, and other characteristics are prominent [37], which would affect farmer enthusiasm to engage in farmland transfer [38].

China’s farmland transfer market should meet the demands of social and economic development. Further, it is imperative to gradually improve the farmland transfer market across various dimensions, including laws and regulations, market mechanisms, the social security system, and the regulation of market players. This would make farmland transfer an effective means to eliminate rural poverty, promote scale production, improve the level of intensification and realize rural revitalization. Some scholars (e.g., Wang et al. [38] and Jiang et al. [39]) have conducted extensive studies on farmland transfer markets and have developed a series of conclusions linked to regional agricultural economic development. However, they have not suggested a large-scale farmland transfer regulation scheme featuring regional characteristics from the market perspective.

### 3.3. Influencing Factors of Farmland Transfer

In general, the research findings on the factors influencing farmland transfer were based on farmer surveys. Econometric methods were used to select the main influencing factors and to analyze their driving mechanism and effects. In the early stage, studies on influencing factors of farmland transfer were mainly analyzed from a single perspective, such as market supply and demand, property rights, social security, and labor force elements. Tan et al. [40] suggested that the main cause of disorderly farmland transfers came from the market supply, and the impact of market demand was not significant. Zhong et al. [16] found that the stability of farmland property rights and the availability of farmer rights to farmland benefits had a large impact on farmer willingness to transfer farmland. Xu and Kong [41] found that the main obstacle for farmland recipients to obtain long-term property rights was a lack of pension and unemployment insurance in rural areas. The continuous migration of the rural workforce to urban areas has greatly contributed to the development of farmland transfer in China. Lin and Ke [42] pointed out that rural labor migration to the cities provided objective conditions for farmland transfer and scale management. With the decrease in the comparative benefits of agriculture and the significant rise in salaries in the non-agricultural sectors, the labor force in rural areas started to migrate, boosting large-scale farmland transfer [39]. The aforementioned phenomenon was corroborated by the study of Liu and Han [43], which showed that the increase of non-agricultural job opportunities was the core factor influencing the emergence of land transfer in economically active rural areas. The development of farmland transfer in economically developed regions was fast. Xu et al. [44] attributed the reason for this to the promotion of collective economic organizations in villages and governments at all levels as well as the demand of the business sector, which was the driving force for farmers.

The influencing factors of farmland transfer are complex and diverse, and they vary in terms of time and space. Therefore, there is a trend of conducting comprehensive analyses on multiple factors of farmland transfer. By simulating farmland transfers to reduce the possibility of farmland fragmentation, Zhong and Wang [17] suggested that reducing the degree of farmland fragmentation should consider multiple factors, including humanistic and natural factors. Dong et al. [45] concluded that factors such as household labor force, family income source, distance from downtown, non-agricultural industrial development at the village or town level, land quality and road distance encouraged the abandonment behavior of farming by farmers living in the suburbs of large cities. Xu and Guo [46] revealed that the stability of land management rights, education level, profession type, and non-agricultural income had a positive impact on farmland transfer, while age negatively impacted farmland transfer. Lü and Li [47] studied the participation of migrant farmers in farmland transfer and found that the age and educational level of farmers and non-agricultural workers had significant impacts on the participation. He et al. [36] showed that the decision-making behavior of farmers with regard to participation in farmland transfer is influenced by many factors, including family demographics, economic characteristics, land endowment, and social environment. The influencing factors of farmland transfer had significant spatiotemporal differences, causing variances in farmland transfer models and situations in terms of time and place.

Based on extant research findings, farmland transfer is also influenced by factors other than a land property rights system, such as farmland transfer policies, farmland quality and participant conditions. Specifically, it includes human factors such as government protection policies, farmer education levels, rural economic levels and geographical conditions, as well as natural factors such as natural resource endowment of regional land, basic conditions of plowing layer and others (Table 2). Furthermore, farmer self-interest may gradually become an important driving factor for farmland transfer.

### 3.4. Distribution Optimization of Farmland Transfer

Geographers have conducted in-depth research into the spatial distribution of farmland and its changes. They have quantitatively explained changes in the spatial distribution of farmland using models. For example, Inés et al. [48] used a rural-land exploration system that based on the GIS (Geographic Information Science) to study the simulation of the Terra Cha region in the northwestern part of Spain under three target conditions: economic, social, and environmental. They also observed changes in the spatial distribution of farmland and suggested a corresponding regulation scheme to optimize farmland distribution [48]. Additionally, the small-scale empirical statistical models of land use change such as CLUE-S (the Conversion of Land Use and its Effects at Small regional extent) and the intelligent agent-based land-use and land-cover change in the time-space process simulation model (ABM/LUCC) have also been widely used in studies to reveal the influential spatial relation of neighboring farmlands and to optimize farmland distribution [49,50]. However, these experience-based statistical models have not been applied to the study of spatial features of farmland transfer due to its complex spatial distribution. Therefore, extant studies cannot rationally explain the causes of change in distribution of farmland transfer, and it is difficult to obtain large-scale generalized conclusions. Instead, the research in this field can only provide recommendations on a regional scale.

When creating and enforcing farmland regulation policies, clarifying time–space distribution features and variation patterns is essential to ensure the sustainable development of agriculture. Therefore, some scholars began to analyze the spatial pattern of farmland transfer and its evolution pattern with economics, geography and mathematical models based on the study of spatial layout of farmland. Bao et al. [51] argued that the status quo of economic development was the cause for variations in scale, method, and rate of farmland transfer between regions. However, the specific spatial distribution patterns of farmland transfer under different models were insufficiently studied [51]. Based on farmer survey data, Bian et al. [12] revealed farmer behavioral variations in both directions of farmland transfer, in and out. Wang et al. [13] believed that the spatial heterogeneity of influencing factors caused the time-space variation features of farmland transfer. Zhang et al. [14] pointed out that the spatial distribution of farmland tenure and transfer was an important basis for developing farmland transfer policies. Liu et al. [15] proposed that the spatial correlation features between itself and the surrounding area cannot be ignored when analyzing the drivers of farmland transfer in a certain area. Although the above studies covered the spatial effects and occurring mechanisms of farmland transfer, they did not discuss the spatial distribution of the scale, direction, and rate of it. Therefore, they are not truly studies on the spatial distribution of farmland transfer. Jin et al. [19] deduced the spatial distribution of farmland transfer directions and patterns in the North China Plain through the ELL model to reveal the characteristics of farmland transfer patterns. However, their study lacked time-series change patterns because their research findings only included the spatial distribution for one year and did not reflect the regional features of farmland transfer, such as rate, popularity, and spatial correlation [19].

As mentioned above, existing studies could describe farmland transfer distribution through quantitative models. However, the lack of studies on the dominant spatial features such as the direction, scale and rate of farmland distribution has led to insufficient scientific knowledge of spatial distribution patterns of farmland transfer. The extant research was thus unable to completely explain the reasons for the changes in the geographical distribution of farmland in a rational manner [19]. The influences of farmland transfer distribution on food security varies due to the spatial differences in regional human and natural elements. It is impossible to fully reveal the scientific aspects of farmland transfer by conducting studies on farmland transfers only in terms of public policies, market mechanisms and influencing factors. Furthermore, it is also difficult to put a comprehensive strategy for the regulation of farmland transfer.

### 3.5. Practices of Farmland Transfer

The practices of farmland transfer reflect the specific content and models of farmland transfer practices, which are demonstrated comprehensively through various farmland transfer models concerning public policies, market mechanisms, influencing factors, spatial distribution, etc. The formulation and development of farmland transfer models are closely related to the national policy system and regional market mechanism, influencing factors, farmland resource features, and so on. The farmland transfer patterns, spatial distribution and subject characteristics also vary considerably among different models. The rapid development of farmland transfer in China has formed diverse models of farmland transfer practices. There are intersections and overlaps between the models and methods of farmland transfer practice. Therefore, research on this topic has gradually shifted from the original cognition of farmland transfer methods to farmland transfer models. Qian [52] divided farmland transfer models into six types: sub-contract, reverse-contract, becoming a shareholder, rent, entrust, and replace. Liu [53] also divided farmland contracted management rights into various transfer models, including sub-contract, lease, assignment, becoming a shareholder, pledge, and inheritance, among others. Although the abovementioned studies are not core transfer models or practices, they represent the organizational process of farmland transfer model contract or cooperation. Sub-contract, lease, assignment, swap, reverse-contract, and becoming a shareholder are farmland transfer models mentioned in Measures for the Administration of Contracted Management Rights of Rural Land. Therefore, it makes sense to use farmland transfer methods as a basis for farmland transfer practice, and it is also consistent with the existing state of research on farmland transfer.

**Figure 2:**
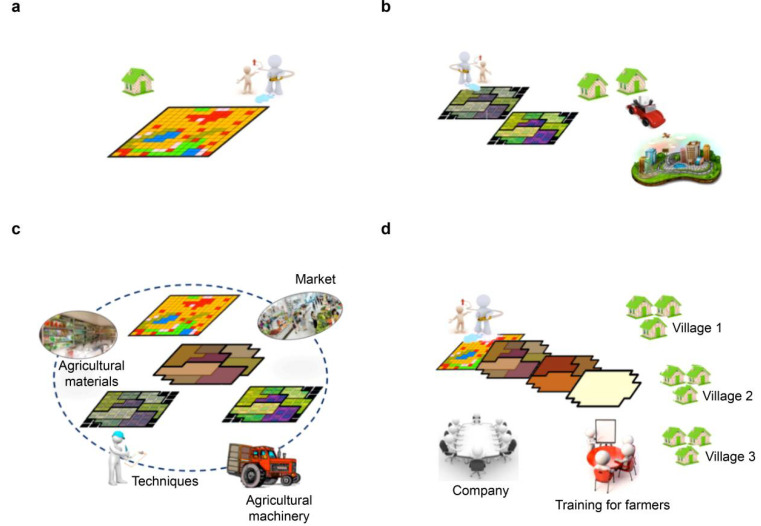
Typical Model Summarizing Farmland Transfer in Practice. (**a**) the “one household manages multiple lands” model, (**b**) the “one farm managed by several households” model, (**c**) the “agricultural cooperative” model and (**d**) the “company + base + farmer” model.

Based on a farmer survey in an administrative village, Huang and Wang [54] proposed the “individual farmer–agent–large farm” land transfer model. Taking the premise that farmland transfer is non-agricultural, Yi and Chen [55] categorized the farmland transfer practice into the leading enterprise-driven model, scale and centralized management model by large farms, and the collective management model by the specialized cooperative. Gao et al. [56] found five typical models of farmland transfer in China: the government-led model, featuring “exchange homestead for house and social security”; the farmer-led model, featuring “market-driven transfer of collectively owned construction land”; the land management rights comprehensive reform model, featuring “authentic right and empowerment”; the scale management model of renting farmland; and the cooperation model based on contractual land as shares. Liu [57] divided the farmland transfer model into the direct transfer model with the contractor as the main player and the indirect transfer model with the participation of the government and agents. Based on the different receivers of farmland transfer, the models were categorized as follows: agricultural business, farmer cooperative, and individual farmer [58,59]. Overall, the current farmland transfer practice models can be categorized into three typical types in the context of national rural revitalization strategy, precise poverty alleviation and modern agricultural system construction. This classification is a comprehensive consideration of the direction, scale, rate and efficiency of farmland transfer from the rural, agricultural and farmer perspectives. The three typical models are: the “one household manages multiple lands” model(Figure 2a) along with the “one farm managed by several households” model (Figure 2b), with farmers as the main agents of transfer; the “agricultural cooperative” model (Figure 2c) with the community as the main player of transfer; and the “company + base + farmer” model, with both farmers and the community as main agents of transfer (Figure 2d). There are significant differences in the main agents of transfer these are degree of scale management, production methods, and geographical spatial forms.

Studies related to farmland transfer practices have analyzed the effects of transfer practice models from different perspectives, and they can provide a reference for the intensified and scaled management of farmland after the transfer. The promotion of farmland transfer models in different regions must adapt to local conditions, so as to promote the scientific use of farmland. Existing research lacks risk analysis of different transfer models. Future studies should be bolstered in this regard to improve risk prevention and an alert mechanism for farmland transfer, thereby ensuring that farmers enjoy the social and economic benefits brought by farmland management rights and promoting the sustainable development of farmland transfer.

## 4. Prospects of Farmland Transfer Studies in China

After decades of development, farmland transfer studies in China have made great progress. However, there are still some gaps in the existing research due to the influence of the land system and technical conditions. It is mainly manifested in the following aspects: First, the uneven socio-economic development and the inconsistent natural conditions of different villages make the farmland transfer conditions vary greatly between regions, thus limiting the depth of the current study. Therefore, the current research findings cannot be implemented in different regions. Second, the study of farmland transfer is extensive and involves many aspects such as urbanization, food security and environmental protection. Therefore, it is necessary to conduct comprehensive analysis based on multiple objectives and consider spatial features of farmland transfer to evaluate how farmland transfer indirectly affects the rights of the main stakeholder through rural development and urbanization. Third, the theoretical system of related studies is not well developed, and there are some limitations such as isolated research findings and lack of innovation. Few studies examine issues from the perspective of geographical patterns and features, thus lacking a comprehensive analysis of the influence processes and rationale for farmland transfer. Based on the key aspects and shortcomings of existing studies, future research on farmland transfer in China should consider the following new topics.

(1) Improving the theoretical system for farmland transfer against the backdrop of systemic reform. Farmland transfer is an economic issue involving regional governments, villages, farmers, agricultural capital owners, investors, managers, and other players. With the rural revitalization campaign and urban-rural integrated development, China’s demand for a rural-land transfer mechanism and systemic development are ever-increasing. Therefore, the key issues of future empirical studies on rural-land transfer may include improving the farmland transfer system framework, enhancing the farmland management rights system, and standardizing the unified platform for the farmland transfer market. Under the current system, theoretical guidance is limited. Therefore, it is necessary to conduct more in-depth discussions about farmland transfer factoring into systemic reform.

(2) Optimizing farmland transfer distribution in line with national strategic decisions. The farmland transfer issue involves various aspects such as the targeted alleviation campaign and the establishment of the modern agricultural model, all of which are important manifestations of the national strategy. Therefore, it is highly relevant in theoretical discussions and empirical studies to depict spatiotemporal variation trends and the spatial distribution of farmland transfer. In addition, it is also important to identify the factors influencing the spatial pattern of farmland transfer and analyze their drivers on the evolution of it. There is also a need to explore the spatial optimization pattern of farmland transfer and to propose specific regulatory measures. In a sense, the spatial distribution of farmland transfer and its evolution mechanism are gradually becoming key links in today’s scientific research on farmland transfer.

(3) Deepening the model for farmland transfer supported by big data. Comprehensive studies should be carried out using empirical analysis, qualitative and quantitative methods to study the scale, popularity, and spatial correlation of farmland transfer supported by big data. Farmland transfer is a social phenomenon and a geographical activity. Big data is more suitable for studies about phenomena and activities with dominant spatial correlations, and the key lies in the building of new models. Integrated models such as spatial econometrics and geo-statistics are more flexible and scientific in summarizing data and analyzing results of farmland transfer. In recent years, the use of these models in various fields has improved model parameters. Therefore, they are more capable of revealing relevant issues of farmland resource management and promoting sustainable farmland transfer.

(4) Applying comprehensive analyses and findings from a cross-disciplinary perspective. Studying the spatial distribution of farmland transfer and its evolution mechanism provides a platform for the comprehensive application of knowledge in various fields, including geographic science, management science, and information science. Carrying out analyses on the spatial distribution of farmland transfer and its evolution mechanism can reveal the spatial and temporal distribution characteristics and patterns of farmland transfer. Furthermore, it is possible to find solutions for farmland administration and policy measures suitable for the region by revealing the evolution mechanism of spatial distribution, thus meeting the scientific information requirements for farmland transfer management in China.

## Figures and Tables

**Table 1:** Important farmland transfer policies at the national level.

Time	Policy Document	Highlights of the Content
September 1992	*Notice on the Opinion on Strengthening the Management of Agricultural Contract* by the State Council	Implementing the household responsibility system with remuneration linked to output is a basic, stable, long-term policy of the CPC for rural areas.
November 1993	*Policies and Measures on the Current Development of Agriculture and Economic Growth in the Rural Areas* by the CPC Central Committee and the State Council	Further improved the household responsibility system with remuneration linked to output, accelerated rural economic development, and maintained social stability in rural areas.
December 1994	*Opinions on Stabilization and Improvement the household responsibility system with Remuneration Linked to Output* by the Ministry of Agriculture	A series of measures, including strengthening the management of the agricultural contract; achieved real stabilization of land contract relations in rural areas.
August 1997	*Notice on Further Stabilization and Improvement of Land Contract Relations* by CPC Central Committee and State Council	Implemented CPC policy that extended the land contract period to effectively protect and mobilize farmers.
December 2001	*Notice on Accomplishing the Transfer of Contract Land Use Rights for Farmers* by the CPC Central Committee	Farmland transfer served as important support for stabilizing and improving land contract relations in rural areas.
May 2002	*Notice on Implementing the CPC Central Committee’s Commitment on Accomplishing the Transfer of Rural-Land Contractual Management Rights for Farmers* by the Ministry of Agriculture	The transfer of rural-land contractual management rights is a very significant achievement in terms of protecting farmer rights, promoting rural development, and maintaining social stability.
August 2002	*Rural-Land Contract Law was passed* by the National People’s Congress Standing Committee	Ensured the long-term stability and improvement of the household responsibility system and firmly protected the land rights of farmers.
November 2003	*Regulations on Certificate of Rural-Land Contractual Management Rights of the People’s Republic of China* by the Ministry of Agriculture	Protected the contractor’s contractual management rights obtained in accordance with the law and strengthened the management of certificate of contracted farmland use rights.
October 2004	*Decision on Deepening the Reform and Tightening Land Management* by the State Council	In keeping with the prerequisites of the plan, the construction land collectively owned by farmers of villages, towns, and administrative towns could be transferred in accordance with the law.
January 2005	*Regulation on the Transfer of Rural Land Contractual Management Rights* by the Ministry of Agriculture	Provided a concrete legal basis for regulating the transfers of rural land contractual management rights and safeguarding the legitimate rights of the two parties involved in the transfer.
January 2008	*Notice on Strictly Enforcing Laws and Policies on Collectively Owned Construction Land in Rural Areas* by the State Council	Strictly controlled the scope of the transfer of construction land collectively owned by farmers, whose management rights should not be used for sale or assignment.
December 2008	*Notice on Accomplishing the Work of Management and Services for the Current Transfer of Rural-Land Contractual Management Rights* by the Ministry of Agriculture	Strengthened the management and services for the transfer of rural-land contractual management rights, stabilizing and improving the basic land management system of rural areas.
June 2009	*Law of People’s Republic of China on Mediation and Arbitration of Disputes over Rural-Land Contract and Management* by the National People’s Congress	Resolved disputes over rural-land contract and management in a fair and timely manner, protected the legitimate rights of the parties involved, and promoted the economic development and social stability of rural areas.
November 2013	*Decisions on Important Issues concerning the Deepening of Reform on All Fronts* by the CPC central committee	Established a unified market for construction land in rural and urban areas; under the prerequisites of meeting the planning requirements and usage control, collectively owned construction land could be registered under the city.
November 2014	*Opinions on Guiding the Orderly Transfer of Rural-Land Management Rights and Development of Scale Management of Agriculture in a Proper Manner* by the CPC central Committee and the State Council	Guided the orderly transfer of rural land (referring to contracted farmland) management rights and the development of scale management of agriculture in a proper manner.
January 2015	*Opinions on Pilot Works of Rural-Land Expropriation, Collectively Owned Construction Land Registration under the City and Homestead System* by the CPC central Committee and the State Council	Improved the land expropriation system, established the registration of collectively-owned rural land for construction under the city, reformed and improved the rural-land system, and established a value-added revenue distribution mechanism to balance the needs of various parties.
January 2015	*Opinions on Guiding the Sound Development of the Transaction Market for the Transfer of Rural Property Right* by the State Council	Under the prerequisites of upholding and refining the basic management system in rural areas, it focused on regulating transfer activities and improved service functions.
January 2015	*Comprehensive Implementation Program for Deepening Rural Reform* by the CPC central Committee and the State Council	Deepened the reform of the rural-land contractual management system, further regulated land management rights, and enabled the orderly transfer of management rights.
July 2016	*Operation Regulation for the Transaction Market for the Transfer Rural-Land Management Rights (Trial)* by the Ministry of Agriculture	Absorbed the transaction procedures of the current transfer market of land management rights, following the order of “application–transaction–contract–service”.

**Table 2:** The Driving Mechanism of the Main Influencing Factors of Farmland Transfer.

Main Influencing Factors		Farmland Transfer-In		Farmland Transfer-Out	
		Direction	Magnitude	Direction	Magnitude
Humanistic Elements	Proportion of non-agricultural population in farmers	−	H	+	H
	Highest education level of the family	+	H	+	L
	Farming income proportion in family income	+	L	+	H
	Arable land per person	−	L	−	L
	Distance to roads	+	L	+	H
	Farmer’s age	-	H	+	L
Natural Elements	Output potential of arable land	+	L	+	H
	Multiple crop index of the arable land	−	L	+	H
	Annual precipitation	+	H	+	L
	Slope of arable land	−	L	−	L
	Thickness of the plowing layer	−	H	−	H
	Organic matter content of the soil	+	H	−	L

Note: + indicates positive impacts, − indicates negative impacts; H indicates a high degree of impact, L indicates a low degree of impact.

## Data Availability

Not applicable.

## References

[1] Jia S., Zhang J. (2006). The Analysis of the Gray Land Market in the Land Allocation System. China Soft Sci..

[2] Hu C. (2010). Meditation and Policy Recommendation on the Amelioration of the Land Banking System. China Land Sci..

[3] Wu W., Tang H., Yang P., Zhou Q.B., Chen Z.X., Shibasaki R. (2010). Model-Based Assessment of Food Security at a Global Scale. Acta Geogr. Sin..

[4] Qu F., Heerink N., Wang W. (1995). Land administration reform in China: Its impact on land allocation and economic development. Land Use Policy.

[5] Gengaje R.K. (1992). Administration of farmland transfer in urban fringes: Lessons from Maharashtra, India. Land Use Policy.

[6] Ye J., Jiang Y., Feng L. (2006). Investigation and Research on Farmland Transfer in China—Analysis and Suggestions Based on the Survey of 17 Provinces in 2005. China Rural Surv..

[7] Zhang Z. (2002). The Development of Farmland Transfer Market in China and Its Way. Chin. Rural Econ..

[8] Huang C., Huang X., Peng C., Zhou Z., Teng M., Wang P. (2019). Land use/cover change in the Three Gorges Reservoir area, China: Reconciling the land use conflicts between development and protection. Catena.

[9] Chen R., Huang C. (2021). Landscape Evolution and Its Impact of Ecosystem Service Value of the Wuhan City, China. Int. J. Environ. Res. Public Health.

[10] Jiang L. (2017). Research on Formation Mechanism and Related Countermeasures of Supply-Side Financing Restraints on New-Type Agricultural Management Entities.

[11] Wang Y. (2015). Study on the Mechanism of the New Agricultural Subject Cultivation under the Background of the Rural Household Differentiation.

[12] Bian Q., Zhou S., Yi X., Wang Y. (2011). Analysis on Status, Characteristics and Regional Variation of Farmland Transfer: An Example of Zhejiang Province. Resour. Sci..

[13] Wang Y., Cai Y., Li H. (2015). The Status of Farmland Transfer in the Context of Spatial Heterogeneity and Its Influencing Factors: Case Studies in Wuhan, Jingmen, and Huanggang. China Land Sci..

[14] Zhang W., Sun D., Yu J., Zhou L., Li H. (2011). Rural Land Management Right and Its Transfer Survey Based on 3S Technologies. Trans. CSAE.

[15] Liu T., Li H., Sun D., Jiang W., Zhou L. (2012). Effects of Regional Land Uses on Agricultural Land Use Right Transfer with Logistic and Auto Logistic Models: A Case in Changping District, Beijing. Sci. Geogr. Sin..

[16] Zhong T., Huang X., Kong P. (2005). Land Property Rights and Farmer Households Willingness of Farmland Leasing. China Land Sci..

[17] Zhong F., Wang X. (2010). Can Land Transfer Markets Reduce Land Fragmentation Currently? Evidence from XingHua City of Jiangsu Province and Bin County of Hei Longjiang Province. Issues Agric. Econ..

[18] Han C., Zhang D. (2018). Transfer Model of Agricultural Land of Market Operation. Inner Mong. Soc. Sci..

[19] Jin G., Deng X., Chen D., Wang P., Sun Z. (2016). Trends and Spatial Patterns of Land Conversions in the North China Plain. Resour. Sci..

[20] Pan C., Chen X. (1997). Thoughts on the Order of Farmland Transfer. Mod. Law Sci..

[21] Wang X. (1993). Discussion on the Mechanism of Farmland Transfer. Chin. Rural Econ..

[22] Li M. (2006). Discussion on the Reform of Rural Land Circulation System and the Construction of New Socialist Countryside. J. Anhui Agric. Sci..

[23] Hu L. (1995). The Effective Transfer of Farmland Faces Six Major Obstacles. China Land.

[24] Jiang X., Li S., Li Q. (2007). Land System Reform and National Economic Growth. Manag. World.

[25] Xia Z. (2014). Suppositional Authentic Right: New Rural Land Circulation System Innovation. J. Nanjing Agric. Univ. (Soc. Sci. Ed.).

[26] Guo J. (1999). Study on the Legal Issues of the Transfer of Farmland Use Right. Tribune Pol. Sci. Law.

[27] Zhang A. (1999). Study on Rural-Urban Land Conversion Transfer and Land Value Increase in Urban and Rural Eco-Economic Interlaced Areas. J. Huazhong Agric. Univ. (Soc. Sci. Ed.).

[28] Kung J.K.S. (2000). Common Property Rights and Land Reallocations in Rural China: Evidence from a Village Survey. World Dev..

[29] Kung J.K. (2002). Off-Farm Labor Markets and the Emergence of Land Rental Markets in Rural China. J. Comp. Econ..

[30] Tian C., Jia S. (2004). Land Tenure, Tenure Security and the Development of Farmland Rental Markets: Theory and Evidence from Jiangsu, Zhejiang and Shandong Provinces. Econ. Res. J..

[31] Lu W., Zhu Z. (2007). An Empirical Analysis of the Relationship Between Supply and Demand of Farmland Transfer—Taking Shanghai as an Example. Chin. Rural Econ..

[32] Jin S., Deininger K. (2009). Land Rental Markets in the Process of Rural Structural Transformation: Productivity and Equity Impacts from China. J. Comp. Econ..

[33] Feng S., Heerink N., Ruben R., Qu F. (2010). Land Rental Market, Off-Farm Employment and Agricultural Production in Southeast China: A Plot-Level Case Study. China Econ. Rev..

[34] Deng X., Zhang S., Hou H. (2010). Problems in Farmland Transfer Market and Countermeasures for Improvement—Based on Empirical Analysis of Chenzhou City, Zhejiang Province. Nat. Resour. Econ. China.

[35] Wen S. (2014). Farmland Transfer: Dilemma and Way Out. Stud. Law Bus..

[36] He X., Jiang T., Guo L. (2016). An Research on the Development of China’s Farmland Transfer Market and the Behavior of Farmers’ Transfer of Farmland—Based on the Survey Data of Farmers in 29 Provinces from 2013 to 2015. Manag. World.

[37] Mou Y., Guo Z. (2006). Game Theory Analysis of Rural Land Conversion Market. Sci. Technol. Manag. Land Resour..

[38] Wang P., Han X. (2009). The Path Choice of Making the Farmland Transfer Market Active-Based on the Construction of a Rural Social Security Legal System. Comm. Res..

[39] Jiang S., Su Q. (2013). Rent Gap in Agricultural Land Circulation and Its Causes. Issues Agric. Econ..

[40] Tan S., Qu F., Nick H. (2003). Analysis of the Causes of the Fragmentation of Land and Its Influencing Factors. China Rural Surv..

[41] Xu Z., Kong X. (2010). Empirical Analysis on the Factors Affecting Transfer Period of the Transferred Land—Based on the Perspective of Transferring Farmers’ Income and Risk. J. Agrotech. Econ..

[42] Lin Z., Ke D. (2006). Deepening the Reform of Farmland System: Motivation, Constraints, and Countermeasures. Shanghai J. Econ..

[43] Liu Y., Han H. (2008). Analysis of the Correlation Between Farmers’ Income and the Transfer of Farmland Use Right. Res. Financ. Econ. Issues.

[44] Xu X., Jiang W., Ying F. (2002). Analysis of the Motivation of Farmland Transfer in China. Manag. World.

[45] Feng Y., Dong Y., Liu Y. (2010). Study on the Characteristics of Farmland Circulation and Its Influencing Factors in Suburbs of Large Cities Based on Farmers’ Survey–A Case Study of 467 Households in Panyu District, Guangzhou. Resour. Sci..

[46] Xu H., Guo Z. (2011). Theoretic and Empirical Research on Influential Factors of Rural Land Transfer Based on the Perspective of Hierarchy Differentiation and Property Rights Preference. China Popul. Resour. Environ..

[47] Lu S., Li H. (2011). The Status Quo and Influencing Factors of quasi-Citizens’ Participation in Cultivated Land Transfer—Based on the Investigation of quasi-Citizen Groups in the Central Region. Chin. Rural Econ..

[48] Inés S., Rafael C.M., David M.B. (2008). GIS-Based Planning Support System for Rural Land-Use Allocation. Comput. Electron. Agric..

[49] Castella J.C., Verburg P.H. (2007). Combination of Process-Oriented and Pattern-Oriented Models of Land-Use Change in a Mountain Area of Vietnam. Ecol. Modell..

[50] Batisani N., Yarnal B. (2009). Uncertainty Awareness in Urban Sprawl Simulations: Lessons from a Small US Metropolitan Region. Land Use Policy.

[51] Bao Z., Xu Z., Gao S. (2009). Regional Differences and Influencing Factors of Rural Land Transfer—Taking Jiangsu Province as an Example. Chin. Rural Econ..

[52] Qian L. (2002). The Main Mode of Land Use Rights Transfer and Issues Needing Attention. World Surv. Res..

[53] Liu W. (2013). Mechanism of Rural Land Transfer in China.

[54] Huang Z., Wang P. (2008). Farmland Transfer and Its Impacts on the Development of Modern Agriculture: Status, Problems, and Solutions. J. Zhejiang Univ. (Humanit. Soc. Sci.).

[55] Yi X., Chen Y. (2010). Analysis of Influencing Factor of Farmers’ Transfer to Cultivated Land and Its “Non-Grain” Planting Behavior and Scale—Based on Survey Data of Farmers in Zhejiang and Hebei Provinces. China Rural Surv..

[56] Gao W., Li Z., Yu L., Wang J. (2010). Speed up Development of Agricultural Informatization and Improve Construction of Agricultural Modernization. Res. Agric. Mod..

[57] Liu L. (2010). A Comparative Study on the Performance of Different Rural Land Transfer Modes.

[58] Wu C. (2012). A Comparative Analysis on the Efficiency Among Different Models of Farmland Transfer. Acad. Res..

[59] Zeng F., Zeng C., Wen X. (2012). Theoretical Model and Mechanism Construction of Farmland Transfer.

